# Etiology, diagnosis and treatment of lumbar disc degeneration: a focus on the mechanism of action of Piezo1 and research perspectives

**DOI:** 10.3389/fcell.2026.1679466

**Published:** 2026-02-13

**Authors:** Yan Gong, Zhaojun Cheng, Jiahui He, Yanchi Gan, Xiaobing Jiang, Jintao Liu

**Affiliations:** 1 Department of Orthopedic Surgery, Suzhou TCM Hospital Affiliated with Nanjing University of Chinese Medicine, Suzhou, China; 2 Nanjing University of Chinese Medicine, Nanjing, China; 3 Department of Spinal Surgery, Second Affiliated Hospital of Guangzhou Medical University, Guangzhou, China; 4 Department of Orthopedics Surgery, The Affiliated TCM Hospital of Guangzhou Medical University, Guangzhou, China; 5 Department of Orthopedics Surgery, Ruikang Hospital Affiliated with Guangxi University of Chinese Medicine, Nanning, China

**Keywords:** lumbar disc degeneration, Piezo1, mechanotransduction, calcium, extracellular matrix

## Abstract

Lumbar disc degeneration (LDD) is a chronic degenerative disease caused by the interaction of genetic and environmental factors, which mainly leads to lower back pain. Its early-stage lesions are insidious and lack reliable biomarkers, and the current diagnosis relies on imaging. Piezo1, a mechanosensitive ion channel widely expressed by intervertebral disc cells, is a core molecule that senses mechanical signals. As a core load-bearing structure of the spine, the mechanical responsiveness of the intervertebral disc is critical for homeostasis, and abnormal mechanical loading is a key trigger of LDD. Piezo1 is deeply involved in the pathology of LDD by sensing abnormal stress and mediating Ca^2+^ influx. On the one hand, activation of the Piezo1-Ca^2+^ axis triggers inflammatory pathways such as NF-κB and the upregulation of proinflammatory factors and matrix-degrading enzymes (e.g., MMPs, ADAMTS); on the other hand, it inhibits the synthesis of the extracellular matrix (ECM), such as type II collagen and proteoglycans, and promotes apoptosis and senescence. The “hypersensitive state” of degenerated discs, in which Piezo1 expression and activity are significantly elevated, is the core link between the transduction and amplification of mechanical signals and pathological cascades. By systematically reviewing the structure and function of Piezo1 and its regulatory mechanism in LDD, we aimed to clarify its role as a core mechanosensing molecule in degeneration and provide theoretical basis for new prevention and treatment strategies.

## Introduction

Lumbar disc degeneration (LDD) is a common spinal disorder triggered by factors such as aging, mechanical stress, and lifestyle, often manifesting as lower back pain, disc herniation, and lumbar instability (Kos et al., 2019). Accelerated deterioration of LDD with age significantly reduces patients’ quality of life and imposes a considerable socioeconomic burden (Kang et al., 2023; Li Z. et al., 2025). The intervertebral disc consists of a proteoglycan-rich nucleus pulposus (NP) and a collagen-rich annulus fibrosus (AF) surrounded by a cartilage endplate (CEP), which together provide structural support and impact absorption of mechanical loads (Song et al., 2024). Given the increasing prevalence of LDD in all age groups and its significant impact, elucidating the molecular mechanisms driving its development is crucial. Current therapeutic approaches, including pharmacological, rehabilitative or surgical treatments, are aimed mainly at relieving pain, but none of them are effective at preventing pathological progression or promoting tissue regeneration (Isa et al., 2022; Mizuno et al., 2024). Therefore, a deep understanding of the pathomechanisms of LDD is key for the discovery of new therapeutic targets and the development of more effective therapies.

Notably, the role of mechanical stress in the development of orthopedic diseases such as LDD and osteoarthritis has been increasingly emphasized (Liu et al., 2024; Xiang Q. et al., 2024). The mechanosensitive cation channel Piezo1 plays a central role as a key mechanoreceptor in mediating cellular responses to mechanical stimuli (Lai et al., 2022). As discovered by Patapoutian’s team in 2010, this channel senses mechanical stimuli and mediates the inward flow of cations (mainly Ca^2+^) across the membrane, converting mechanical signals into chemical signals, triggering cellular excitation and initiating downstream signaling pathways (Coste et al., 2012). Piezo1 has a conserved sequence structure across mammals (Coste et al., 2010). Cryoelectron microscopy studies revealed the formation of homotrimers with a unique three-bladed propeller-like conformation (Zhao et al., 2018). This structure enables efficient conversion of mechanical stresses to electrochemical signals through “blade” bending, “cap” rotation and ion gating mechanisms (Yang et al., 2022). Piezo1 is widely involved in many important physiological processes, such as bone formation, vascular development, blood pressure regulation and innate immunity (Guo et al., 2023).

In recent years, Piezo1 has been recognized as a key mechanically activated ion channel in the pathogenesis of LDD. Numerous studies have shown that Piezo1 is abnormally activated in degenerated disc tissues and is deeply involved in the pathology of LDD (Guo et al., 2023). Inhibiting or interfering with Piezo1 channel function effectively delays the progression of LDD under experimental conditions, suggesting that Piezo1 may be an important potential target for combating this disabling disease (Li F. et al., 2025). Therefore, comprehensively studying and elucidating the specific mechanism and function of Piezo1 in LDD is highly important. The aim of this study was to review the biological functions, expression and regulatory mechanisms of Piezo1 in intervertebral disc tissues, with a focus on its role in the pathogenesis of LDD, and to discuss the latest research advances on its role as a potential therapeutic target.

## Concept of LDD

LDD is a progressive, pathologic process of structural and functional deterioration of the intervertebral discs that occurs in the lumbar spine region (Saleem et al., 2013). The core pathology is a complex cascade of biomechanical, biochemical, metabolic, and cell biological responses of the disc tissue (including the nucleus pulposus, annulus fibrosus, and cartilaginous endplates), resulting in a progressive loss of its normal biological functions (load bearing, shock cushioning, and maintenance of spinal mobility) (Risbud and Shapiro, 2014). Although this process can be considered part of spinal aging, it is often significantly accelerated or exacerbated by genetic predispositions, the accumulation of mechanical loads, an impaired nutrient supply, and an increased inflammatory response, ultimately transcending the realm of purely age-related physiologic changes and evolving into a pathological condition (Kirnaz et al., 2022; Kang et al., 2023).

## Core features of LDD

**FIGURE 1:**
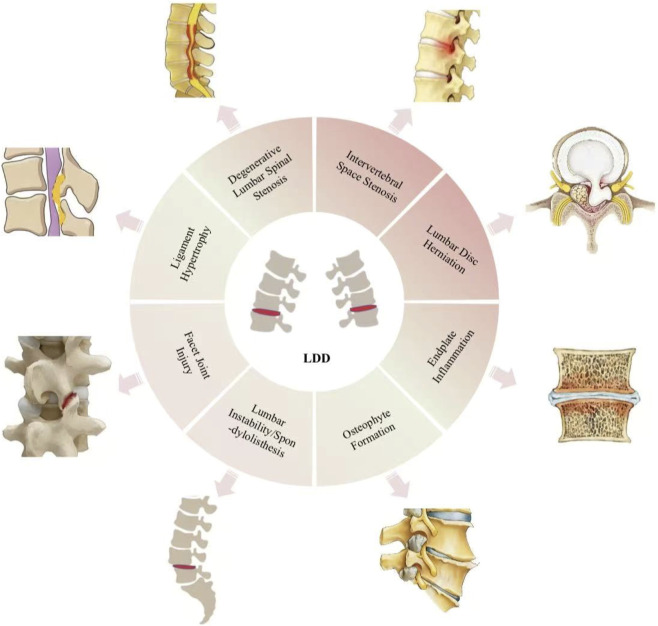
Key structural manifestations and pathological context of LDD. The illustration depicts the progressive structural breakdown characteristic of LDD, including dehydration and shrinkage of the NP, fissure formation in the AF, and sclerosis or calcification of the CEP. These manifestations typically follow a progression from biochemical NP changes to macrostructural AF and CEP failures, with severity correlating with the stage of degeneration. Clinically, early NP dehydration often underlies discogenic pain managed conservatively, while advanced AF fissures (especially with herniation) or severe CEP sclerosis are frequently associated with radiculopathy or refractory pain that may necessitate surgical intervention. Critically, the aberrant biomechanical stress and inflammatory microenvironment generated by all these structural changes are potent activators of the Piezo1 channel. Thus, the resulting dysfunction and cellular stress collectively represent the core pathological context in which Piezo1-mediated mechanotransduction drives disease progression, making this pathway a promising therapeutic target across the degeneration spectrum.

The core pathological process of LDD begins with a significant reduction in the content of key proteoglycans in the medulla, resulting in a loss of water-binding capacity and a sharp decrease in water content, causing the medulla to collapse from its hydrated gel state, dry out and shrink, and impair core hydraulic buffering function (Song et al., 2024). Moreover, the replacement of type II collagen with type I collagen in the nucleus pulposus matrix and the abnormal proportion, cross-linking, and alignment of type I collagen in the annulus fibrosus collectively lead to decreased disc elasticity, increased brittleness, and deterioration of mechanical properties (Lian et al., 2017; Zeldin et al., 2020). This is accompanied by a decrease in the number and activity of myeloid and fibrocyclic chondrocyte-like cells, which have a diminished ability to synthesize healthy extracellular matrix (ECM) and abnormally elevated activities of catabolic enzymes (MMPs, ADAMTS), resulting in accelerated degradation of the ECM and disruption of tissue homeostasis (Liang et al., 2022; Zhang et al., 2022). In addition to cartilage endplate calcification, hardening and capillary network reduction or occlusion caused by the obstruction of nutrient pathways severely hinder the diffusion of nutrients and metabolic waste removal, resulting in a vicious cycle of aggravated cellular dysfunction and matrix decomposition (Sun et al., 2024; Yang H. et al., 2024). Eventually, the cumulative effect of the above changes manifests itself in macrostructural damage, such as narrowing of the intervertebral space due to loss of nucleus pulposus height, fibrous annulus tears, and modic changes in subendoplasia (Figure 1), which severely weakens the ability of the discs to withstand and distribute loads (axial, shear, and torsion), leading to loss of biomechanical stability of the lumbar spinal segments, an increase in abnormal stresses, and acceleration of the secondary degeneration of neighboring structures (Ashinsky et al., 2021; Corporal Michael, 2021).

## Epidemiology of LDD

Lumbar disc degeneration is highly prevalent, and its development is closely related to age, occupation, lifestyle and other factors (Samanta et al., 2023). LDD is essentially a pathological process highly associated with aging (Chiu et al., 2024). Imaging evidence (MRI-demonstrated black discs, height loss, bulges, etc.) increases dramatically with age in the asymptomatic population (Wocial et al., 2021). A systematic review and meta-analysis revealed that MRI manifestations of disc degeneration were more prevalent in adults with low back pain (especially those 50 years of age and younger) than in asymptomatic controls and that there was a strong correlation between the two, with manifestations present in approximately 30% of asymptomatic individuals and approximately 50% of symptomatic individuals (Brinjikji et al., 2015a). However, Brinjikji et al. (2015b) demonstrated that multiple imaging manifestations of degenerative spinal disease are highly prevalent in asymptomatic individuals and increase with age, mostly as a result of the normal aging process, and do not consistently correlate with low back pain; therefore, interpretation needs to be combined with the prevalence in asymptomatic people of the same age to differentiate between normal and pathologic changes. The lower lumbar spine (especially the L4–L5 and L5–S1 segments) is the most common site of lumbar disc herniation because it has the highest mechanical load, high mobility, high axial pressure on the spine, and a complex biomechanical environment in the lumbosacral junction area, which is the area where degeneration is the most common and serious (Buser et al., 2021). Low back pain is the leading cause of disability worldwide, with LDD being one of its primary underlying cause (Isa et al., 2022). Low back pain is a common symptom affecting individuals across all income levels and age groups worldwide. Between 1990 and 2015, disability-adjusted life years attributable to this condition increased by 54% (with the most pronounced rise occurring in low- and middle-income countries) (Hartvigsen et al., 2018). It is now the leading cause of disability globally. Most patients present without identifiable aetiology, with only a minority exhibiting pathological factors such as vertebral fractures. High-risk groups include manual labourers and smokers. New-onset cases often recover rapidly but are prone to recurrence, Some cases progress to persistent disabling low back pain (associated with factors like initial severe pain), with the associated healthcare burden varying by country and projected to rise further. The challenge is greater in low- and middle-income countries, necessitating enhanced research and global collaboration (Koes et al., 2006). Therefore, unravelling the molecular mechanisms underlying LDD pathogenesis and exploring early intervention strategies are crucial for reducing disability rates.

## Causes of LDD

The pathogenesis of LDD is not driven by a single factor but rather by the deeply intertwined and synergistic effects of multiple factors, such as genetic susceptibility, environmental exposure, biomechanical abnormalities, and dysregulation of complex molecular signaling networks, which together initiate and accelerate the process of progressive disc structural and functional deterioration (Samanta et al., 2023).

### Internal factors

Genetic susceptibility: A large body of evidence suggests that genetic factors are central to LDD susceptibility. Battié et al. (Battié et al., 2008) demonstrated that identical twins have a high degree and pattern of disc degeneration even in different environments, indicating that genetics contributes to approximately 74% of the difference in the risk of the disease. People with a family history of LDD have a significantly greater risk of developing the disease, and this strong heritability stems from key gene variants (Martirosyan et al., 2016). Specific variants in collagen genes (e.g., COL9A2 and COL11A1) impair the synthesis, structure, or cross-linking of collagen types I, II, IX, and XI, directly weakening the tensile strength and toughness of the fibrous ring and increasing the risk of tearing under mechanical loading (Noponen-Hietala et al., 2003). Mutations in the proteoglycan core protein-encoding gene (ACAN) impair the synthesis and stability of aggregated proteoglycans in the medulla, leading to a decrease in their hydrophilicity and charge-fixing ability, resulting in a decline in the “hydraulic damping” function of the medulla and a loss of cushioning capacity (Stattin et al., 2022). Imbalanced mutations in genes encoding matrix metalloproteinases (e.g., MMP-3 and MMP-13) and their inhibitors disrupt the balance of extracellular matrix metabolism, triggering excessive degradation of collagen and proteoglycans and accelerating matrix destruction (Vo et al., 2013). These genetic variants significantly lower the biological threshold for an individual disc to resist stress and maintain homeostasis, making carriers susceptible to initiation, faster progression, and greater severity of degeneration in response to environmental factors such as strain, obesity, and smoking.

Age-related biological decline: Aging is the most significant nonmodifying risk factor for LDD, which is essentially a systematic decline in the biological function of intervertebral disc cells, manifested by the progressive senescence of intervertebral disc cells, including telomere shortening, mitochondrial dysfunction leading to the accumulation of reactive oxygen species (ROS), decreased repair capacity, and increased apoptosis (Zhao et al., 2007; Vo et al., 2016; Wen et al., 2022). Along with cellular senescence, the anabolic capacity of the ECM is significantly weakened, whereas the catabolic tendency is increased, disrupting the critical balance that maintains tissue homeostasis (Wu Y. et al., 2022). In addition, reduced vascularization and the inherent low-oxygen, low-nutrient microenvironment within the intervertebral disc further deteriorate with age, exacerbating cellular dysfunction (Mavrogonatou et al., 2023).

### Dysregulation of molecular signaling networks (intrinsic molecular mechanisms)

Beyond genetic and aging factors, the dysregulation of intricate molecular signaling networks constitutes a fundamental intrinsic layer of LDD pathogenesis. Activation of the inflammatory response: Mechanical injury, cellular stress, or death releases damage-associated molecular patterns (DAMPs) that activate local and infiltrating immune cells (e.g., macrophages) in the intervertebral disc, leading to sustained high expression of key proinflammatory factors (e.g., TNF-α and IL-1β) (Gawri et al., 2014; McGill University Health Centre, 2014; Alkhatib et al., 2014). These factors form a positive feedback loop that strongly stimulates the production of catabolic enzymes and exacerbates the degradation of proteoglycans and collagen while inhibiting the expression of matrix synthesis genes and promoting the release of pain mediators (e.g., substance P, prostaglandins), which are directly associated with clinical symptoms (Song et al., 2024).

Oxidative stress injury: In aging, inflammatory and ischemic-hypoxic environments, there is an overproduction of ROS in the intervertebral disc, which exceeds the scavenging capacity of endogenous antioxidant systems (e.g., superoxide dismutase (SOD) and glutathione) (Feng et al., 2017; Wen et al., 2022). Excessive ROS directly oxidatively damage proteins, lipids and DNA, leading to cellular dysfunction, accelerated senescence and death, and activation of proinflammatory signaling pathways (e.g., NF-κB) and catabolic pathways, forming a vicious cycle (Guo et al., 2021).

Abnormalities in mechanical signaling: Intervertebral disc cells sense mechanical stimuli in the microenvironment through mechanosensitive ion channels (e.g., Piezo1 and TRPV4), the expression or function of which is often dysregulated during LDD (Gao et al., 2022; Palmer et al., 2025). Aberrant mechanical signaling can misregulate intracellular calcium homeostasis, inflammatory gene expression, and extracellular matrix remodeling-related genes, preventing cells from adapting to physiological loads and instead interpreting normal or mildly aberrant mechanical stimuli as damage signals that drive degeneration-related gene expression (Walter et al., 2011; Vergroesen et al., 2015). Sustained aberrant activation of Piezo1 channels has been shown to promote inflammatory responses and catabolism (Sun et al., 2020).

### External factors

Abnormal mechanical loading: Long-term repeated abnormal mechanical stimulation is a core extrinsic driver of accelerated LDD. A sedentary lifestyle leads to prolonged high-pressure static loading of the lumbar intervertebral discs, limiting nutrient diffusion (Song et al., 2024). High peak stresses from heavy labor and shear forces tend to cause the accumulation of microdamage in the fiber loops (Guo et al., 2025). Obesity significantly increases sustained axial loading in the lumbar spine, accelerating disc water loss and structural collapse (Lundon and Bolton, 2001). Moreover, severe spinal trauma can lead to direct structural damage to the intervertebral disc, triggering an inflammatory cascade and disrupting the local biomechanical environment, providing a “launching pad” for the degenerative process (Park et al., 2013; Galbusera et al., 2014).

Unhealthy lifestyle: Nicotine, carbon monoxide and other toxins in tobacco smoke have strong vasoconstrictive effects, which significantly reduce capillary blood flow in the endplate region, impeding the delivery of nutrients to the central part of the intervertebral disc and the removal of metabolic waste (Elmasry et al., 2015). Nicotine also directly induces apoptosis and inhibits matrix synthesis in intervertebral disc cells (Akmal et al., 2004). Moderate physical activity helps promote disc nutrient exchange and maintain peripheral spinal muscle strength to provide dynamic stabilization (Malandrino et al., 2014). Chronic physical inactivity is detrimental to these protective mechanisms.

### The dual role of mechanical stress and the Piezo1 threshold

Collectively, external factors such as abnormal loading and unhealthy lifestyles converge to alter the mechanical microenvironment of the disc. It is crucial to distinguish between the physiological and pathological roles of mechanical stress in LDD. Moderate, dynamic loading (e.g., from walking and appropriate exercise) is essential for maintaining disc cell metabolism, promoting nutrient exchange, and stimulating ECM synthesis—these represent physiological mechanical stimuli (Malandrino et al., 2014; Song et al., 2024). Conversely, when the magnitude, frequency, duration, or pattern of loading exceeds the tissue’s adaptive and reparative capacity, it transforms into pathological mechanical stress. This transition hinges on the cell’s ability to decode mechanical signals, with mechanosensitive ion channels like Piezo1 acting as a critical molecular switch that discriminates between adaptive and detrimental stimuli (Vergroesen et al., 2015; Lai et al., 2022). The following section details the molecular cascades initiated when this switch is erroneously engaged by pathological stress.

## Diagnosis of LDD

The diagnosis of LDD is based on multidimensional clinical integration, which centers on clarifying the causal associations between degenerative structures and clinical symptoms, as well as ruling out underlying diseases such as infections, tumors, and inflammatory spondylopathies. (Liyew, 2020). Through systematic evaluation of symptoms, signs, imaging, and necessary ancillary tests, the degree of degeneration, degree of nerve compression, and source of symptoms can be clarified, providing guidance for individualized treatment (Saleem et al., 2013). In the clinical assessment, symptoms are characterized by low back pain (aggravated by activity, relieved by rest, and may persist in the chronic phase), radicular symptoms (radiating pain, sensory abnormalities, and motor deficits in the lower extremities), and neurogenic claudication (pain/weakness in the lower extremities after walking, relieved by rest) (Isa et al., 2022). Physical examination includes nerve root tone tests, such as the straight leg raise test; neurologic function tests, such as sensory/muscle strength/reflex evaluations; and localized tenderness tests, such as paraspinal tenderness (Van Der Windt et al., 2010).

Imaging and ancillary diagnostic techniques are key supports for LDD diagnosis (Sąsiadek and Jacków-Nowicka, 2023). Radiographs may reveal bony structural degeneration, such as narrowing of the intervertebral space, endplate sclerosis/osteosclerosis, and force line abnormalities, but do not allow for direct assessment of disc herniation or nerve compression (Reunanen et al., 2023). MRI, as a diagnostic gold standard, can be used to assess degenerative manifestations such as reduced signals in the nucleus pulposus (“black disc”), fibrous annulus fissures, Modic changes in the endplates, and the relationship between disc herniation and neural structures, as well as to identify abnormalities of the cauda equina through multiplanar soft-tissue imaging (Waldenberg et al., 2018). CT is advantageous for 3D reconstruction of bony encumbrances, bony stenosis of the spinal canal, calcified protrusions, and complex deformities (Brinjikji et al., 2015b). In terms of ancillary techniques, stimulated discography can identify the “responsible segment” in multisegmental degeneration (Pinto et al., 2022). Laboratory tests (blood tests, CRP, HLA-B27, etc.) are used to rule out infections and inflammatory diseases. In contrast, neurophysiological examination can localize nerve damage and differentiate root damage from peripheral neuropathy (Lolis et al., 2018). Overall, the diagnosis of LDD needs to be symptom-oriented and MRI-centered, combining dynamic clinical assessment with targeted adjuncts.

## LDD treatment

The treatment of LDD focuses on relieving symptoms, slowing the progression of degeneration, and restoring spinal function and should be individualized according to the patient’s age, degree of degeneration, symptomatic characteristics, and functional needs, with emphasis on the synergy of stepped interventions and etiological prevention (Ohnishi et al., 2022).

### Conservative treatment: multi-tool synergy and integration of Chinese and western medicines

Conservative treatment, as the first choice for most patients with early or mild LDD, emphasizes the synergistic use of multiple means to achieve symptom control and functional improvement, in which, in addition to conventional Western medical therapies, the integration of traditional Chinese medicine and physical interventions can further increase the efficacy of treatment (Sturm et al., 2024). Moreover, pharmacological treatment needs to be a combination of Chinese and Western medicines and nonsteroidal anti-inflammatory drugs through the inhibition of cyclo-oxygenase to reduce the levels of inflammatory mediators, such as prostaglandins, and rapid pain relief, but the course of treatment needs to be controlled to reduce the risk of gastrointestinal ulcers and cardiovascular events (Yang S. et al., 2024). Neurotrophic agents, on the other hand, improve nerve root symptoms such as numbness and sensory abnormalities in the lower limbs by promoting nerve myelin synthesis and are particularly suitable for patients with concomitant nerve damage (La Binch et al., 2014).

### Physical intervention: stratified implementation and synergistic application of acupuncture and massage

Physical interventions need to be implemented at different levels, and acupuncture and massage are important supplements. In the acute stage, to relieve inflammation and edema at the core, in addition to low-frequency pulsed electrotherapy, ultrasound and other modern means of physical therapy, acupuncture can be selected from the kidney, large intestine, commissioning, and ring jump acupuncture points, through the regulation of local qi and blood flow, the inhibition of neural excitability to alleviate pain, the acute stage of the use of millipercenter needle shallow puncture, avoiding strong irritation, and the remission stage of traction therapy (applicable to mild to moderate disc herniation, with spinal stenosis or the acute inflammatory period prohibited) can be combined with massage. In the relief period, on the basis of traction therapy (applicable to mild to moderate disc herniation, spinal stenosis or acute inflammation is prohibited), combined with Tuina massage, the gentle kneading and rolling method is the main method, with a focus on relaxing spasmodic muscles of the lumbar and dorsal back, with pressure at the Ashi point, lumbar Yangguan and other acupoints to improve the local circulation, and attention should be given to avoiding the violence of rotary wrenching or heavy pressure to prevent aggravation of disc herniation or nerve injury (Xin et al., 2022).

### Rehabilitation and lifestyle adjustment: core muscle training and overall protection

Rehabilitation exercises are centered on core muscle group training, such as bridge exercise (to strengthen the lower back muscles) and plate support (to strengthen the transverse abdominal muscles), and need to strictly follow the “step-by-step, painless principle”, avoiding stooping and weight-bearing movements. Lifestyle adjustments, such as weight reduction, avoiding sedentary activities, and choosing a hard mattress to maintain the physiological curvature of the lumbar vertebrae, are also crucial to reduce the accelerating factors of degeneration. Reducing the accelerating factors of degeneration and combining Chinese and Western medicines in a conservative program through multitarget interventions can not only quickly relieve symptoms but also take into account overall conditioning, which provides a more comprehensive treatment option for patients with early-stage LDD (Jeong et al., 2015).

### Surgery: indication control and principles of selection of surgical procedures

Surgery is suitable for patients with ineffective conservative treatment, obvious nerve compression (e.g., decreased muscle strength, urinary and fecal dysfunction) or segmental instability, and the indications need to be strictly grasped. Nucleus pulposus removal can remove the protruding nucleus pulposus through endoscopic technology, quickly relieving nerve compression, with the advantages of less trauma and faster recovery, making it suitable for patients with simple herniated discs (Kwon and Moon, 2025). Interbody fusion, on the other hand, is used to stabilize the diseased segment through pedicle screw fixation combined with implant fusion in cases of spinal stenosis, slippage, or instability. Although it can eliminate the source of pain, it may increase the risk of degeneration of adjacent segments and should be used with caution in young patients (Walsh et al., 2020). Artificial disc replacement, as an alternative to fusion, preserves segmental motion function by implanting a bionic prosthesis and reduces stress concentrations in neighboring segments and is now mainly indicated for young and middle-aged patients with single-segment degeneration and no obvious instability; however, more follow-up data are needed to support long-term safety (Shedid et al., 2005).

### Emerging biological therapies: frontier directions for delaying degeneration

Emerging biologic therapies offer new directions for slowing or reversing degeneration and are mostly in the experimental or early clinical exploration stage. Stem cell transplantation can repair degenerated disc tissues by differentiating into nucleus pulposus-like cells and secreting extracellular matrix components such as type II collagen. Small-sample studies have shown that stem cell transplantation can significantly improve pain scores, but the cell source, transplantation method, and long-term tumorigenicity still need to be optimized (Urits et al., 2019). Platelet-rich plasma (PRP) injections utilize platelet-released growth factors (e.g., PDGF and TGF-β) to promote fibrous annulus repair in patients with discogenic pain (Pirvu et al., 2014). The development of mechanosensitive channels (e.g., Piezo1) that target drugs focuses on regulating the pressure sensing and metabolic balance of the intervertebral disc, which has been shown to inhibit the release of inflammatory factors and slow the degeneration of the nucleus pulposus; thus, Piezo1 is expected to become a new type of drug for early intervention in the future (Shi et al., 2022).

Overall, the treatment of LDD should follow the principles of “conservative prioritization, surgical precision, and biological therapeutic exploration” and take into account the individual differences of patients to achieve the multiple goals of “symptomatic relief, functional recovery, and delayed degeneration”.

## Structural composition of Piezo1

Piezo1 is a large transmembrane protein, and elucidation of its structure is crucial for understanding the functional mechanism of its mechanosensitive channel. The protein has a large molecular weight and no obvious sequence homology with known ion channels or proteins (Saotome et al., 2018). Cryo-electron microscopy revealed that Piezo1 contains 2,547 amino acid residues, which assemble into a homotrimer. The overall structure presents a unique three-bladed propeller shape (Ge et al., 2015). Each subunit contains approximately 38 transmembrane helices, and the entire trimer contains a total of 114 transmembrane helices, which together form a “nanowheel” configuration (Zhao et al., 2018). This configuration induces local lipid bilayer deformation into a dome shape, thereby mediating mechanically induced channel opening (Guo and MacKinnon, 2017).

**FIGURE 2:**
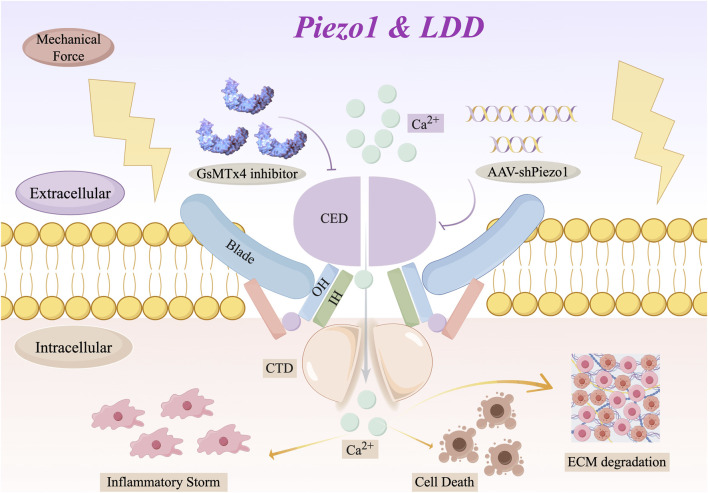
Structural domains of Piezo1 and their therapeutic implications for LDD. (By Figdraw). This illustration details the core functional modules of the Piezo1 channel–including the force-sensing Blades, the ion-conducting pore lined by Inner (IH) and Outer Helices (OH), and the gating-regulatory C‐terminal Extracellular (CED) and Intracellular (CTD) Domains–to inform rational therapeutic strategies for DDD. Specifically, the extracellular blade/CED region represents a target for direct pharmacological inhibition (e.g., by the peptide GsMTx4), whereas upstream gene-silencing approaches (e.g., AAV-shPiezo1) aim to reduce overall channel expression. By mapping these distinct interventions onto Piezo1’s structure, this figure highlights its unique tractability as a target for mechano-based therapy in disc degeneration, moving beyond generic description to guide disc-specific therapeutic design.

Structurally, each subunit of Piezo1 contains a central ion-conducting pore module and three peripheral mechanical transduction modules. Notably, the central ion-conducting pore module is covered by an extracellular cap domain (Wang et al., 2018; Fang et al., 2021). The central ion-conducting pore is composed of an outer helix, an inner helix, a cytoplasmic C-terminal domain (CTD), and an extracellular C-terminal domain (CED) that determines unit conductance, ion permeability, and selectivity (Qin et al., 2021) (Figure 2). The peripheral mechanical transmission module includes a long beam-shaped structure, peripheral blades, and a unique anchoring structure domain (Zhao et al., 2019). The long beam structure supports the leaves and bridges them to the central hole module, whereas the large extracellular leaf structure domain allows the plasma membrane to bend, and the three leaves assemble to form a functional trimer (Fang et al., 2021). In addition, the anchoring domain formed by the hairpin structure connects to the CTD plane via inner and outer helices, maintaining the integrity of the channel (Zhao et al., 2018). Functional studies have shown that the amino acid residue located at position 1–2190 of the mechanical transduction module is critical for conferring mechanical sensitivity to the trimeric channel pore (Zhao et al., 2016). Overall, the trimeric structure of Piezo1 and the precise arrangement and interaction of its various domains enable it to synergistically sense mechanical stimuli and convert them into electrical signals, thereby activating ion conduction and fulfilling its core function in mechanotransduction (Qin et al., 2021).

## Biological function of Piezo1

The gating and functional regulation of Piezo1 channels directly depend on changes in cell membrane tension (De Vecchis et al., 2021). When different types of mechanical forces, such as tensile force, fluid shear force, or matrix mechanical stimulation, cause changes in membrane tension, this channel is activated and opens, mediating the inward flow of cations such as K^+^, Na^+^, Ca^2+^, and Mg^2+^ (especially those with high selectivity for Ca^2+^) across the membrane (Zhao et al., 2018). These changes in ion flow can activate downstream signaling pathways, thereby regulating key biological processes such as cell proliferation, differentiation, and apoptosis (Tang et al., 2022). Piezo1 is widely expressed in various mammalian tissues and cells and participates in the regulation of numerous important physiological and pathological processes (Pathak et al., 2014).

In the cardiovascular system, as a key mechanotransduction molecule, Piezo1 is highly expressed specifically in endothelial cells and is indispensable for embryonic vascular development (Jin et al., 2024). Studies have shown that loss of Piezo1 function leads to abnormal arrangement of embryonic endothelial cells and cardiovascular developmental disorders. Moreover, its abnormal activation can cause pathological changes in endothelial and smooth muscle cells, promote vascular remodeling, and participate in pathological processes such as hypertension and atherosclerosis (Ranade et al., 2014). In the hematopoietic system, Piezo1 regulates red blood cell morphology by mediating Ca^2+^ influx. Its mutation not only affects the membrane stability and deformability of mature red blood cells in patients with hereditary spherocytosis (HS) but also significantly interferes with multiple stages of red blood cell production, primarily through abnormal mechanical signal transduction and Ca^2+^ homeostasis imbalance (Albuisson et al., 2013; Jankovsky et al., 2021). In the field of oncology, Piezo1 plays a key role in angiogenesis, cancer cell proliferation and migration, and immune regulation by transducing mechanical signals in the tumor microenvironment (Yu and Liao, 2021; Zhao et al., 2022). Its abnormally high expression in various malignant tumor tissues, such as liver cancer, stomach cancer, prostate cancer, and breast cancer, can significantly increase the proliferation activity, invasive ability, and metastatic potential of cancer cells, thereby accelerating tumor progression (Dombroski et al., 2021). In the renal system, Piezo1 acts as an important mechanical signal hub, participating in physiological processes such as maintaining the glomerular filtration barrier, regulating water and salt reabsorption in the renal tubules, and sensing renal vascular tension (Li et al., 2022). In pathological conditions, abnormal Piezo1 activation is involved in processes such as chronic kidney disease fibrosis and vascular remodeling in hypertensive nephropathy (Yuan et al., 2024). In summary, Piezo1 plays a key regulatory role in the pathological processes of various diseases (cardiovascular, hematological, oncological, and renal diseases). In-depth exploration of the mechanical signal transduction mechanism mediated by Piezo1 will not only help elucidate the pathogenesis of related diseases but also provide an important theoretical basis and potential molecular targets for the development of new intervention strategies.

## Piezo1 in context: comparison with other mechanosensitive and Ca^2+^-permeable channels in the disc

While disc cells express various Ca^2+^-permeable channels (e.g., TRPV4, Piezo2) to sense their microenvironment, a critical question arises: what uniquely positions Piezo1 as a therapeutic target in LDD? Channels like TRPV4 are known to respond to diverse stimuli, including osmotic changes and inflammatory mediators, contributing to disc cell mechanobiology and pathophysiolog (Gao et al., 2022; Palmer et al., 2025). In contrast, converging evidence highlights Piezo1’s non-redundant, central role in pathological mechanotransduction.

Piezo1’s uniqueness is underscored by four key aspects. First, it is a dedicated, high-sensitivity mechanosensor for a broad spectrum of pathological stresses (compression, shear, stiffness) relevant to LDD (Vergroesen et al., 2015; Lai et al., 2022). Second, its activation triggers substantial Ca^2+^ influx, potently driving high-amplitude pathways like NF-κB and NLRP3 (Sun et al., 2020; Wang et al., 2021). Third, its expression dynamically correlates with degeneration severity, a feature less consistent for other channels (Wang et al., 2021; Shi et al., 2022). Finally, and most compellingly, specific Piezo1 inhibition—notably more than targeting other channels—robustly attenuates inflammation, matrix degradation, and cell death in LDD models (Sun et al., 2020; Liu et al., 2023; Lin et al., 2025). Thus, Piezo1 acts as a critical upstream hub, justifying its focused targeting to disrupt a central axis of degeneration.

## Piezo1-mediated mechanotransduction: from physiological sensing to pathological activation

Piezo1 serves as the primary molecular sensor for disc cells (NPCs, AFCs, and CECs) to perceive their complex mechanical microenvironment, responding to compression, tension, shear, and fluid flow (Wang et al., 2021; Liu et al., 2023). Under moderate, cyclic physiological loads, transient Piezo1 activation mediates controlled Ca^2+^ influx, a signal that acts as a second messenger to promote anabolic pathways, contributing to tissue homeostasis, ECM maintenance, and normal disc function (Wu J. et al., 2022). The shift to pathology occurs when mechanical stimuli become excessive or aberrant. *In vitro* studies indicate that prolonged static compression, high-frequency oscillatory loads, or supraphysiological shear stress can lead to sustained, rather than transient, Piezo1 channel opening, resulting in pathological Ca^2+^ overload (Sun et al., 2020; Wang et al., 2021). This “signal transformation” is governed by a context-dependent functional threshold, determined by the integration of mechanical load parameters, ECM stiffness, and the local inflammatory milieu (Walter et al., 2011; Shi et al., 2022). When the combined cues surpass this threshold, Piezo1-mediated signaling tips from adaptive regulation to a sustained pathological driver. This breach initiates a vicious cycle: for instance, ECM stiffening promotes sustained Piezo1 activation, which upregulates matrix-degrading enzymes and pro-inflammatory factors via pathways like NF-κB and NLRP3 (Sun et al., 2020; Wang et al., 2021). Subsequent ECM degradation further alters the mechanical microenvironment, leading to more Piezo1 activation—a self-perpetuating “biomechanical-biological” feedback loop that accelerates degeneration (Vergroesen et al., 2015).

Key mechanisms of Piezo1 in LDD—such as its role in NLRP3 inflammasome activation (Sun et al., 2020), mitochondrial fission via the Ca^2+^/CaMKII/Drp1 axis (Lin et al., 2025), and the induction of ferroptosis (Xiang Z. et al., 2024)—have been robustly demonstrated using *in vitro* models. However, these models cannot fully replicate the complex, multidirectional, and dynamic mechanical microenvironment of the *in vivo* disc. Therefore, while core pathways are well-supported, the precise *in vivo* mechanical thresholds and the detailed choreography of some proposed positive feedback loops require further validation in more complex models.

## Critical synthesis of methodological approaches and unresolved challenges

As summarized in Table 1, the causal link between aberrant mechanical loads and Piezo1 dysregulation is supported by diverse methodologies, each presenting distinct advantages and interpretive challenges. *In vitro* models (e.g., cyclic stretch (Sun et al., 2020), hydrostatic pressure (Shi et al., 2022)) isolate quantifiable forces to directly prove Piezo1’s mechanosensitivity, while stiffness models (Wang et al., 2021) simulate the consequence of ECM degradation. In contrast, *in vivo* models introduce essential biological complexity, making it difficult to isolate the primary mechanical signal from secondary inflammation—a key methodological challenge. Despite this diversity, the findings converge into a cohesive pathophysiological sequence: excessive force/stiffness → Piezo1 over-activation → pathological Ca^2+^ influx → downstream inflammatory, apoptotic, and ferroptotic cascades. The consistent observation of these core Piezo1-mediated pathways across disparate models reinforces their robustness, while highlighting that the relative contribution of immediate mechanotransduction versus secondary inflammation is model-dependent. Future studies employing *ex vivo* organ culture under controlled load with targeted cytokine blockade may best disentangle this mechanical-chemical interplay.

**TABLE 1:** Methodological approaches investigating Piezo1 activation by mechanical load and its role in driving inflammatory and catabolic responses in LDD.

Model type	Representative study	Load method/Parameters	Inflammatory/Catabolic markers studied	Piezo1’s role in inflammation/Catabolism (key findings)	Primary limitation
*In Vitro* (Cellular)	Sun et al. (2020)	Cyclic stretch (20%, 0.1Hz; human NP cells)	IL-1β, TNF-α, NLRP3, ASC, caspase-1	Activates NLRP3 inflammasome & IL-1β maturation via Ca^2+^/NF-κB; reversed by siRNA/GsMTx4	Single mechanical stimulus
*In Vitro* (Cellular)	Shi et al. (2022)	Compressive stress (1.5%/15% intensity, Flexcell)	LC3-II/I, Bax/Bcl-2, MMP-13	Disrupts autophagy/apoptosis balance, upregulates MMP-13; alleviated by GsMTx4	Lacks complex *in vivo* load
*In Vitro* (Cellular)	Wang et al. (2021)	Substrate stiffness (1/25 kPa hydrogels; human NP cells)	SA-β-gal, ROS, caspase-3	Mediates stiffness-induced senescence/apoptosis via Ca^2+^/ROS/ER stress; reversed by silencing	Static culture, no dynamic load
*In Vitro* (Cellular)	Ke et al. (2023)	Matrix stiffness (1/12/25 kPa plates; human NP cells)	Drp1 (p-Ser616), Apoptosis markers	Induces mitochondrial fission and apoptosis via Ca^2+^/ERK1/2/Drp1 axis	2D culture, lacks 3D microenvironment
*Ex Vivo* (Organ Culture)	Sun et al. (2022)	Single axial impact (rat spinal segments, *ex vivo* culture for 14 days)	IL-1β, MMP-9, MMP-13, NLRP3, ATP, lactate	A single impact injury without structural disruption upregulates Piezo1, which mediates NLRP3 inflammasome activation, IL-1β & MMPs expression, and abnormal energy metabolism. Silencing Piezo1 attenuates this response	*Ex vivo* model, lacks systemic factors and long-term *in vivo* environment
*In Vivo* (Animal)	Liu et al. (2023)	Lumbar instability surgery (rat L4/L5)	Cleaved caspase-3, TNF-α, IL-6	Drives AF cell apoptosis via Ca^2+^/Calpain2/Caspase3 axis and promotes inflammation	Models post-traumatic instability
*In Vivo* (Animal)	Xiang et al. (2024b)	Caudal disc needle puncture (mouse)	Lipid peroxidation, GPX4	Mediates mechanical stress-induced iron influx and ferroptosis	Cannot fully replicate human biomechanics

## Dynamic evolution and threshold of Piezo1 activation during LDD progression

The expression and activity of Piezo1 are dynamically amplified during LDD progression, correlating with degeneration severity (Wang et al., 2021). This amplification is driven by a degenerative microenvironment featuring both sustained abnormal mechanical stress and elevated pro-inflammatory factors like TNF-α and IL-1β(Shi et al., 2022). In the early phase, Piezo1 acts as the primary mechanotransducer, where moderate upregulation and Ca^2+^ influx may initially represent an adaptive response (Wu J. et al., 2022). However, a reinforcing vicious cycle between mechanical stress, inflammation, and matrix degradation soon establishes, leading to significant and persistent Piezo1 overexpression in the progressive phase (Wang et al., 2021; Shi et al., 2022). At this stage, pathological Ca^2+^ influx through overactive Piezo1 channels directly triggers deleterious cascades, including NLRP3 inflammasome activation, mitochondrial dysfunction, and ferroptosis (Sun et al., 2020; Xiang Z. et al., 2024; Lin et al., 2025). In the late stage, Piezo1 signaling becomes entrenched, driving terminal structural failures such as endplate calcification and annulus fissures. Importantly, such macrostructural damage itself creates a secondary wave of aberrant mechanical and inflammatory stimuli that further amplifies Piezo1 dysregulation in a feed-forward manner.

The shift from moderate to pathological Piezo1 activity is governed by a context-dependent functional threshold. This threshold is not fixed but is determined by the integrated microenvironmental context, including the magnitude/duration of mechanical load (Walter et al., 2011), extracellular matrix stiffness (Wang et al., 2021), and the local inflammatory/oxidative stress milieu (Shi et al., 2022; Lin et al., 2025). This framework clarifies that Piezo1 is the core primary sensor whose activation initiates degeneration; however, its activity is subsequently secondarily amplified by the resulting inflammatory and structural changes, cementing its dual role as both initiator and amplifier. When combined signals exceed this threshold, Piezo1-mediated signaling tips from potential adaptation to sustained pathological driving, underscoring the rationale for therapeutic strategies aimed at modulating Piezo1 or its downstream nodes to interrupt this vicious cycle (Sun et al., 2020; Jia et al., 2024; Lin et al., 2025).

## Pathological mechanism of Piezo1 in LDD

### Impact of structural disruption on Piezo1 dysregulation

The progression of LDD is often accompanied by macrostructural failures such as nucleus pulposus protrusion, annulus fibrosus fissures, and endplate fractures. These breaches compromise compartmental integrity, drastically altering the local mechanical microenvironment. The loss of hydraulic containment increases stress concentration on remaining tissues, while the infiltration of systemic immune cells and cytokines creates a pro-inflammatory milieu. This combined biomechanical and biological insult can further upregulate and sensitize Piezo1 channels, creating a maladaptive feedback loop. Therefore, while Piezo1 activation can initiate degeneration under abnormal loading, structural damage itself becomes a powerful secondary driver that amplifies Piezo1-mediated pathological signaling, accelerating the degenerative cascade.

### Piezo1 promotes degradation of the cartilage endplate

As the core channel for the intervertebral disc nutrition supply and mechanical interface, the structural stability of the CEP is crucial to intervertebral disc homeostasis (Xiang et al., 2022). Human bipedalism subjects the CEP to long-term axial mechanical stress, which, combined with aging and other factors, accelerates CEP degeneration. During degeneration, the CEP exhibits significant oxidative stress and chronic inflammation, accompanied by bursts of ROS and abnormal release of proinflammatory factors, driving ECM degradation and tissue calcification and forming a vicious cycle of “damage-degeneration-redamage” (Kamali et al., 2021). Piezo1 plays a central role in this pathological cascade. It is activated by sensing mechanical stress in the CEP microenvironment, and its activation triggers a large influx of Ca^2+^, initiating a multidimensional degenerative cascade reaction (Jiang et al., 2021). Among these, Ca^2+^ activates calmodulin-dependent protein kinase II (CaMKII), promoting the phosphorylation of the mitochondrial fission factor Drp1, inducing excessive mitochondrial fission, disrupting the mitochondrial membrane potential, inhibiting ATP synthesis, exacerbating ROS production, triggering energy metabolism crises and oxidative damage, and ultimately leading to CEC senescence, apoptosis, and ECM synthesis disorders (Lin et al., 2025a). Lin et al. (2025b) demonstrated that under LPS-stimulated inflammatory conditions, Piezo1 expression was significantly upregulated in CECs, exacerbating mitochondrial dynamics disorders through the aforementioned Ca^2+^/CaMKII/Drp1 axis, forming a positive feedback loop, and significantly accelerating CEC degeneration and death. Moreover, Peng et al. (Peng et al., 2025) confirmed that Piezo1 can also activate downstream effector molecules (Yes-associated protein (Yap)) through nonclassical pathways by promoting Ca^2+^ influx and F-actin polymerization, thereby increasing ROS levels, increasing oxidative stress, promoting ECM degradation enzyme expression, and amplifying inflammatory responses, thereby disrupting CEP structure in multiple dimensions. Ding et al. (2022) confirmed that the homolog of Yap, YAP1, is a key regulatory factor in mechanism-mediated IVDD. Its expression decreases with increasing intensity and duration of mechanical stimulation. The activation of YAP1 can delay CEP degeneration by increasing the promoter activity of ACAN and COL2A1. Additionally, Piezo1 may regulate YAP1 expression through the Hippo signaling pathway. Therefore, targeting YAP1 in conjunction with regulating Piezo1 represents a potential therapeutic strategy for treating CEP degeneration.

Given the central role of Piezo1, targeting its activity or downstream nodes has significant therapeutic potential. Experiments have confirmed that the use of Piezo1 inhibitors (such as GsMTx4) or AAV-shRNA to knockdown Piezo1 expression or the inhibition of downstream molecules (such as the CaMKII inhibitor KN-93 and the Drp1 inhibitor Mdivi-1) can effectively improve mitochondrial function, reduce oxidative stress and inflammation, inhibit apoptosis, and thereby alleviate CEP degeneration (Lin et al., 2025). In summary, Piezo1 constitutes the core regulatory network of CEP degeneration through two pathways: mitochondrial dysfunction mediated by the Ca2+/CaMKII/Drp1 axis and oxidative stress-inflammation-ECM degradation mediated by the F-actin/Yap axis. Targeting Piezo1 and its key downstream nodes (such as CaMKII, Drp1, and Yap) or synergistically regulating the protective potential of YAP1 provides a theoretical and experimental foundation for innovative strategies to delay and treat LDD.

### Piezo1 promotes ferroptosis in intervertebral disc cells

Iron-dependent programmed cell death, characterized by lipid peroxidation and cell membrane damage caused by the accumulation of lipid reactive oxygen species, plays an important role in LDD (Lu et al., 2021). The phospholipid hydroperoxide reductase GPX4 is a key molecule in the defense against ferroptosis; it consumes glutathione to reduce lipid peroxides and maintain membrane stability (Zhang et al., 2021). Research indicates that Piezo1 is a core regulatory hub involved in iron death in LDD (Xiang Z. et al., 2024). In NP cells, abnormal mechanical stress can activate Piezo1 channels located on the cell membrane and endoplasmic reticulum, triggering Ca^2+^ kinetic disorders, which both increase extracellular calcium influx and promote the release of endoplasmic reticulum calcium stores, ultimately leading to intracellular calcium overload (Jia et al., 2024). This calcium signal storm directly triggers endoplasmic reticulum stress, and sustained endoplasmic reticulum stress further amplifies calcium release, forming a vicious cycle. Calcium overload and endoplasmic reticulum stress synergistically significantly inhibit GPX4 synthesis and function, weakening the ability of cells to clear lipid peroxides and ultimately driving ferroptosis (Jia et al., 2024). Moreover, Piezo1 activation can also mediate abnormal iron influx through a nontransferrin receptor (TFRC)-dependent pathway, causing iron ion overload and exacerbating lipid peroxidation damage (Xiang Z. et al., 2024). The dual effects of Piezo1 (inhibiting GPX4 function and inducing iron overload) jointly disrupt iron metabolism homeostasis in NPC, induce ferroptosis, lead to reduced ECM synthesis and increased degradation, and accelerate the progression of LDD.

Selenium supplementation can provide significant protection against ferroptosis caused by Piezo1–GPX4 axis imbalance. Selenium, an essential trace element, acts by integrating into selenoproteins as selenocysteine (Avery and Hoffmann, 2018). Selenium supplementation specifically upregulates the expression of endoplasmic reticulum-resident selenoprotein K (SelK) (Jia et al., 2021). As a key endoplasmic reticulum stress regulator, SelK can alleviate Piezo1-mediated calcium overload and endoplasmic reticulum stress, blocking its vicious inhibitory cycle on GPX4. Moreover, selenium can directly increase the expression and activity of GPX4, improving the antioxidant capacity of cells through the selenium‒GPX4 axis and effectively removing lipid peroxides to resist ferroptosis (Jia et al., 2024). Previous studies have confirmed that selenium supplementation can upregulate the expression of multiple selenium proteins, and the antiferroptotic effect of the selenium‒GPX4 axis has been verified in follicular helper T cells (Ingold et al., 2018). This mechanism also applies to NPC. Selenium supplementation synergistically antagonizes Piezo1-induced ferroptosis through two pathways: on the one hand, it alleviates endoplasmic reticulum stress via SelK, and on the other hand, it directly activates GPX4, thereby protecting NPC function and maintaining ECM homeostasis (Jia et al., 2024). In summary, ferroptosis is an important pathological mechanism underlying LDD caused by mechanical overload, and its occurrence depends on Piezo1-mediated calcium dysregulation, iron overload, and GPX4 inhibition. Targeting Piezo1 (e.g., through gene knockout or the inhibitor GsMTx4) can reverse iron/calcium dysregulation and restore GPX4 function. By enhancing SelK and GPX4 activity, selenium supplementation strategies offer an innovative therapeutic perspective for delaying or even reversing LDD. This pathological axis is further corroborated by *in vivo* findings. For instance, Xiang et al. (2024a) demonstrated in a mouse needle puncture model that Piezo1-mediated iron influx exacerbates NP cell ferroptosis and accelerates disc degeneration.

### Piezo1 promotes the apoptosis and senescence of intervertebral disc cells

The core pathological mechanism of LDD involves the apoptosis and senescence of NPCs and AFCs in an abnormal mechanical environment, with Piezo1 playing a key regulatory role in this process (Wu J. et al., 2022; Liu et al., 2023). Excessive mechanical stress or gradually increasing ECM stiffness during the degeneration process can directly activate Piezo1 channels on the cell membranes of NPC and AFC cells (Lei et al., 2024). In NPC, activation of Piezo1 triggers a multistep cascade: on the one hand, Piezo1 activates the NLRP3 inflammasome by inducing Ca^2+^ influx, promoting the maturation and release of proinflammatory factors such as IL-1β, with downstream signal amplification dependent on the NF-κB pathway (Sun et al., 2020); on the other hand, rigid substrates trigger endoplasmic reticulum stress and oxidative stress through Piezo1, directly driving cellular senescence and apoptosis programs (Wang et al., 2021). Notably, Piezo1 also mediates a self-amplifying pathological cycle. After matrix stiffness activates Piezo1, it promotes the secretion of periosteal proteins, which in turn further activate the NF-κB pathway. Activated NF-κB then upregulates the expression of periosteal proteins, forming a positive feedback loop that accelerates NPC aging (Wu J. et al., 2022). In addition, Piezo1 activation promotes apoptosis through the MAPK and ERK1/2 pathways while triggering unique iron metabolism disorders (Ke et al., 2023). It does not rely on TFRC but directly promotes abnormal iron ion influx, disrupting intracellular iron homeostasis and thereby exacerbating ferroptosis, a novel form of cell death (Xiang et al., 2024b). These processes collectively lead to NPC anabolic disorders, manifested as accelerated ECM degradation and the inhibition of autophagy (Shi et al., 2022).

In the AF region, abnormal mechanical loads can also trigger pathological changes in the AFC through the Piezo1 channel. Upon sensing excessive mechanical stimulation, Piezo1 mediates a massive influx of Ca^2+^, which sequentially activates Calpain2 and Caspase3 — this Piezo1/Ca^2+^/Calpain2/Bax/Caspase3 signaling axis is the core pathway driving apoptosis and aging in AFC (Liu et al., 2023). Like in NPC, sustained activation of Piezo1 also promotes the secretion of inflammatory mediators such as TNF-α, IL-6, and IL-1β by AFCs, further worsening the local microenvironment and forming a vicious cycle of proinflammatory and prodegenerative effects (Liu et al., 2023). Overall, Piezo1, as a core molecule in mechanochemical signal transduction, synergistically promotes inflammatory responses, aging processes, and various forms of cell death (apoptosis, ferroptosis) in intervertebral disc cells through the multidimensional integration of NLRP3 inflammasome activation, NF-κB signaling, the ERK1/2 pathway, ferroptosis induction, and Calpain2/Bax/Caspase3 cascade reactions. The positive feedback loop it mediates between periosteal proteins and NF-κB, along with its stiffness sensitivity, further tightly links biomechanical abnormalities with cellular fate (Wu J. et al., 2022). Since Piezo1 is an upstream hub in the LDD regulatory network, targeting and inhibiting its activity or that of key downstream molecules (such as NLRP3, Calpain2, and the ferroptosis pathway) may block mechanical stress-induced LDD, delay ECM degradation, reverse degeneration, and become a novel therapeutic strategy. The central role of Piezo1 in mechanically induced apoptosis is strongly supported by animal models. Liu et al. (2023) showed that lumbar instability in rats upregulates Piezo1 in AF cells, activating the Ca^2+^/Calpain2/Caspase3 axis to drive apoptosis, and that silencing Piezo1 alleviated this degeneration.

### Piezo1 promotes inflammatory responses in intervertebral discs

The inflammatory cascade of LDD begins with continuous stimulation of the NPC by abnormal mechanical stress, in which the Piezo1 ion channel acts as a core transduction molecule (Wu J. et al., 2022). When ECM stiffness increases or fluid shear stress intensifies, Piezo1 is activated and mediates the influx of cations such as Ca^2+^ and Mg^2+^, triggering a triple pathological cascade. First, intracellular Ca^2+^ overload directly induces endoplasmic reticulum stress and mitochondrial ROS bursts, forming a “Ca^2+^-ROS-endoplasmic reticulum stress” positive feedback loop that drives apoptosis (Yang et al., 2025). Second, Ca^2+^ influx synergizes with mechanical stress to activate the NF-κB pathway, promoting the nuclear translocation of this factor and upregulating the expression of proinflammatory factors such as TNF-α, IL-6, and IL-1β. Moreover, NF-κB forms a self-amplifying loop with periosteal proteins, accelerating cellular senescence (Wu J. et al., 2022). Third, NF-κB synchronously initiates the transcription of key components of the NLRP3 inflammasome (NLRP3, proIL-1β, and proIL-18), laying the foundation for the final effects of inflammation (Guo et al., 2023). Piezo1 expression is positively correlated with the severity of LDD (Wang et al., 2021). Abnormal mechanical stress inhibits ECM synthesis and promotes the expression of catabolic enzymes (MMP-3/13, ADAMTS-4/5), leading to a vicious cycle of ECM degradation→matrix hardening→further activation of Piezo1, which continuously amplifies inflammatory damage. Sun et al. (2020) reported that the Piezo1 inhibitor GsMTx4 or siRNA significantly downregulated mechanical stretch-induced NLRP3, ASC, and IL-1β mRNA expression and effectively inhibited caspase-1 activity and IL-1β secretion, directly verifying its core role in inflammasome activation.

The deeper pathogenic role of Piezo1 lies in its ability to trigger the final burst and self-maintenance of inflammatory signals through the Ca^2+^-NLRP3 axis. During the assembly stage of the NLRP3 inflammasome, Ca^2+^ overload induced by Piezo1 acts as a key secondary stimulus, promoting the successful aggregation of the NLRP3 receptor, ASC adaptor protein, and procaspase-1 precursor into a functional complex in the cytoplasm (Sun et al., 2020). This complex activates caspase-1, which cleaves Gasdermin D to induce pyroptosis and destroy tissue structure while also converting proIL-1β into mature IL-1β and releasing it in large quantities (Fu and Wu, 2023). The latter reactivates NF-κB through autocrine/paracrine pathways, forming a positive feedback loop of “Piezo1→Ca^2+^→NLRP3→IL-1β→NF-κB→NLRP3,” causing inflammation to increase exponentially (Sun et al., 2022). Moreover, IL-1β activates the ERK1/2 pathway, inhibits the synthesis of core ECM components, and induces high expression of matrix degradation enzymes, leading to metabolic collapse of the ECM (Li et al., 2023). Key intervention studies have shown that GsMTx4 or siRNA-mediated silencing of Piezo1 can block mechanical stretch-induced NLRP3 inflammasome assembly, which is the mechanism underlying its ability to inhibit downstream effects (Sun et al., 2020). The calcium chelating agent BAPTA-AM not only inhibits inflammasome activation but also reverses NF-κB nuclear translocation, thereby highlighting the central role of Ca^2+^ (Yan et al., 2023).

Therefore, targeting the Piezo1-Ca^2+^-NLRP3 axis can simultaneously intervene in the initiation, amplification, and tissue degradation processes of inflammation, providing a breakthrough strategy for reversing the pathological process of LDD. The relevance of this Piezo1-NLRP3 axis in a complex tissue context is confirmed by *ex vivo* organ culture models. Sun et al. (2022) reported that a single impact injury to rat spinal segments upregulated Piezo1 and triggered a Piezo1-dependent cascade involving NLRP3 activation and IL-1β release, linking acute mechanical injury to sustained inflammation. Collectively, the pathological roles of Piezo1 in LDD are multifaceted, driving degeneration through distinct yet interconnected molecular cascades in different disc compartments. The key mechanisms summarized in Table 2 highlight its central role as a mechanotransducer that exacerbates CEP degradation, triggers NP cell ferroptosis, promotes apoptosis and senescence of disc cells, and amplifies inflammatory responses, ultimately leading to ECM destruction and disc dysfunction.

**TABLE 2:** Summary of piezo1-mediated pathological mechanisms in lumbar disc degeneration.

Pathological process	Core mechanism involving Piezo1	Key downstream effectors/Pathways	Representative experimental evidence	References
Degradation of Cartilage Endplate	Sensing mechanical stress in CEP microenvironment, leading to Ca^2+^ influx	Ca^2+^/CaMKII/Drp1 axis → mitochondrial fission; F-actin/Yap axis → oxidative stress and inflammation	*In vitro* CEC model under inflammatory stimulus: Piezo1 upregulation exacerbates mitochondrial dysfunction and cell degeneration via Ca^2+^/CaMKII/Drp1. Inhibition of this axis alleviates damage	Lin et al. (2025)
Ferroptosis of Nucleus Pulposus Cells	Mediating mechanical stress-induced Ca^2+^ overload (promoting ER stress) and non-TFRC-dependent iron influx	Inhibition of GPX4 (via ER stress); Intracellular iron overload → Lipid peroxidation	Mechanical overload model in NPCs: Piezo1 activation triggers ferroptosis by disrupting calcium/iron homeostasis and inhibiting GPX4. Selenium supplementation rescues cells via SelK/GPX4	Jia et al. (2024), Xiang et al. (2024b)
Apoptosis & Senescence of Disc Cells	Mediating mechanical stress-induced Ca^2+^ overload (promoting ER stress) and non-TFRC-dependent iron influx	In NPCs: NLRP3 inflammasome/NF-κB pathway; Periostin-NF-κB positive feedback loop. In AFCs: Ca^2+^/Calpain2/Bax/Caspase3 axis	Stretch-induced NP cell model: Piezo1 is essential for NLRP3 inflammasome activation and IL-1β production. AF cell model: Piezo1-mediated Ca^2+^ influx activates the Calpain2/Caspase3 apoptotic cascade	Sun et al. (2020), Wu et al. (2022a), Liu et al. (2023)
Apoptosis & Senescence of Disc Cells	Acting as a core transducer, converting aberrant mechanical signals into pro-inflammatory signaling	In NPCs: NLRP3 inflammasome/NF-κB pathway; Periostin-NF-κB positive feedback loop. In AFCs: Ca^2+^/Calpain2/Bax/Caspase3 axis	Stretch-induced NP cell model: Piezo1 is essential for NLRP3 inflammasome activation and IL-1β production. AF cell model: Piezo1-mediated Ca^2+^ influx activates the Calpain2/Caspase3 apoptotic cascade	Sun et al. (2020), Guo et al. (2023)

## The potential of Piezo1 as a therapeutic target for LDD

**FIGURE 3:**
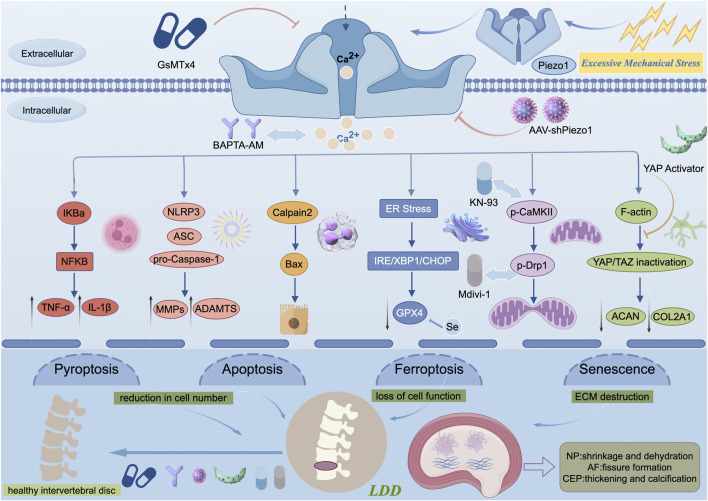
Integrated schematic of Piezo1-mediated signaling cascades in LDD. (By Figdraw). This diagram synthesizes the key pathological pathways initiated by Piezo1 activation under excessive mechanical stress in the intervertebral disc. The illustrated connections represent a temporal and mechanistic cascade: immediate ion flux (Ca^2+^) rapidly activates signaling kinases and proteases (e.g., CaMKII, Calpain2), leading to transcriptional regulation (e.g., via NF‐κB) and cellular organelle stress within hours, which cumulatively drive cell fate decisions (apoptosis, ferroptosis, senescence) and extracellular matrix remodeling over days to weeks. The depicted interactions are supported by multiple lines of evidence detailed in the review, including in vitro mechanostimulation studies, cell-specific pathway analyses, and in vivo degeneration models. Key pharmacological and genetic interventions targeting specific nodes (e.g., GsMTx4, BAPTA‐AM, KN-93, selenium) are indicated, highlighting potential therapeutic strategies to interrupt this integrated degenerative network.

In recent years, as the role of Piezo1 in slowing the progression of LDD has gradually been revealed by the academic community, research in this area has increased (Figure 3). Piezo1, a mechanosensitive ion channel, is significantly expressed in intervertebral disc tissue, and its expression level is positively correlated with the severity of LDD. These characteristics make it a potential target for precision treatment of LDD (Yan et al., 2023). Its core therapeutic potential stems from its clear amenability to intervention: the application of the pharmacological inhibitor GsMTx4 can effectively block NLRP3 inflammasome activation induced by mechanical stress, significantly reduce the release of proinflammatory factors such as IL-1β and TNF-α, and reverse calcium overload-mediated mitochondrial dysfunction and ferroptosis processes. Gene silencing strategies (such as the AAV-shPiezo1 vector) achieve this goal by locally knocking down channel expression, thereby alleviating endplate calcification, inhibiting NPC apoptosis, and correcting iron metabolism disorders (Lin et al., 2025). Most importantly, targeting Piezo1 can simultaneously improve the three core pathological phenotypes of LDD—inhibiting inflammatory storms, restoring ECM synthetic metabolism, and blocking multiple types of programmed cell death (apoptosis, pyroptosis, and ferroptosis). This multieffect creates a unique advantage for single-target comprehensive intervention (Guo et al., 2023). The role of calcium signaling as a hub further reinforces its therapeutic value: the downstream inhibitor BAPTA-AM, by chelating Ca^2+^, can synergistically inhibit NF-κB activation and catabolic enzyme expression, confirming that targeting this pathway can produce multidimensional protective effects (Yan et al., 2023).

The therapeutic potential of Piezo1 has further demonstrated synergistic effects and clinical translation innovations. On the basis of its mechanosensitive properties, combined with biomechanical regulation (such as customized rehabilitation training to reduce abnormal loads), a “mechanical–pharmacological” synergistic intervention model can be formed to reduce the channel activation frequency at the source. At the molecular synergy level, selenium supplementation strategies enhance GPX4 function by upregulating the selenium protein SelK, thereby specifically antagonizing Piezo1-mediated ferroptosis (Jia et al., 2024). Moreover, combined YAP1 activators (targeting the Hippo pathway) can reverse the inhibition of ECM synthesis via the F-actin/Yap axis and promote collagen and proteoglycan regeneration (Peng et al., 2025). To address clinical delivery bottlenecks, leveraging the expression characteristics of Piezo1, the development of targeted delivery systems for intervertebral discs (such as biodegradable hydrogels loaded with GsMTx4 or liposome-encapsulated siRNA) can overcome the avascular barrier of intervertebral discs and enhance drug bioavailability. Despite challenges such as inhibitor permeability optimization and long-term safety assessment, the development of gene therapy (e.g., AAV vectors) and smart responsive materials paves the way for clinical translation.

The diverse therapeutic strategies targeting Piezo1, each with its unique mechanism and stage of development, are comparatively summarized in Table 3. This overview underscores the potential of modulating Piezo1 activity directly or through its downstream nodes, while also acknowledging the practical hurdles that must be overcome for successful clinical application. In summary, Piezo1, with its spatial targeting ability, multidimensional pathological regulation, and broad synergistic intervention window, offers a breakthrough direction for precision treatment of LDD.

**TABLE 3:** Comparison of potential therapeutic strategies targeting piezo1 in LDD.

Therapeutic strategy	Mechanism of action	Experimental outcomes (in vitro/*In Vivo*)	Potential advantages	Current challenges/Limitations	References
Pharmacological Inhibition (e.g., GsMTx4)	Directly blocks the Piezo1 ion channel pore	Reduces NLRP3 inflammasome activation, IL-1β release, and NP cell apoptosis under mechanical stress. Attenuates CEP degeneration	Well-characterized inhibitor; Provides proof-of-concept for target druggability	Poor permeability into avascular disc; Potential off-target effects; Long-term safety unknown	Sun et al. (2020), Lin et al. (2025)
Gene Silencing (e.g., AAV-shPiezo1)	Knocks down Piezo1 expression locally	Attenuates CEP degeneration, improves mitochondrial function, and corrects iron metabolism dysregulation	High specificity; Potential for sustained effect	Delivery efficiency to disc cells; Immune response to viral vector; Safety of long-term knockdown	Lin et al. (2025)
Selenium Supplementation	Upregulates selenoproteins (SelK) and enhances GPX4 activity	Antagonizes Piezo1-mediated ferroptosis, reduces oxidative stress, and attenuates disc senescence	Targets downstream pathological axis (ferroptosis); Potential for dietary/nutraceutical intervention	Optimal dose and formulation for disc; Systemic vs. localized effects; Bioavailability	Jia et al. (2024)
Synergistic Biomechanical Modulation	Reduces abnormal mechanical load, decreasing Piezo1 activation frequency	Customized rehabilitation (core training) is proposed to lower stress on discs, theoretically reducing pathological Piezo1 signaling	Non-invasive; Addresses a root cause (abnormal loading); Can be combined with other therapies	Difficult to quantify precise load reduction; Patient compliance; Requires individualized design	Shi et al. (2022)
Synergistic Biomechanical Modulation	Targets the Hippo pathway to counteract Piezo1/F-actin/Yap axis-mediated ECM synthesis inhibition	Customized rehabilitation (core training) is proposed to lower stress on discs, theoretically reducing pathological Piezo1 signaling	Multi-target approach; Potential to reverse ECM loss	Complexity of pathway crosstalk; Lack of specific IVDD intervention studies; Drug development at early stage	Ding et al. (2022), Peng et al. (2025)

## Summary and outlook

Piezo1, as the core molecular sensor for perceiving mechanical stress in intervertebral disc tissue, is specifically expressed in terms of spatial distribution, and its dependency-activated activation during the degenerative process collectively forms the mechanical signal decoding basis for the pathological cascade of LDD (Guo et al., 2023). Under the combined effects of abnormal mechanical loads and an inflammatory microenvironment, excessive opening of Piezo1 channels triggers pathological Ca^2+^ influx, driving the irreversible progression of LDD through three mechanisms: first, Ca^2+^ overload directly activates downstream catabolic networks, including NLRP3 inflammasome-mediated IL-1β storms and NF-κB pathway-driven overexpression of matrix metalloproteinases, leading to ECM degradation and endplate calcification (Sun et al., 2020); second, an imbalance in the mitochondrial‒iron death axis induces mitochondrial fragmentation and energy crisis through Ca^2+^/CaMKII/Drp1 signaling while triggering iron overload in a non-TFRC-dependent manner, synergizing with GPX4 function inhibition to cause lipid peroxidation and NPC iron death (Lin et al., 2025); third, a mechanical‒metabolic positive feedback loop, increased ECM stiffness further activates Piezo1, which inhibits collagen/proteoglycan synthesis through the F-actin/Yap axis, forming a vicious cycle of “mechanical damage‒channel activation‒matrix destruction” (Peng et al., 2025). This multidimensional pathological effect further reinforces the status of Piezo1 as a key therapeutic target and diagnostic marker for LDD.

Although the central role of Piezo1 in LDD has been confirmed, current research still faces three limitations. First, the mechanism of spatial mechanical signal decoding remains poorly understood. Existing findings have focused primarily on Ca^2+^ signaling at the cellular level, but the mechanical response specificity of heterogeneous regions within the intervertebral disc (such as the CEP-NP interface) and how mechanical load parameters (frequency/amplitude/duration) dynamically regulate Piezo1 conformation activation remain poorly understood. Second, the translation of targeted intervention tools is limited. Inhibitors such as GsMTx4 can effectively block pathological cascade reactions, but they are constrained by the low permeability barrier of the avascular intervertebral disc structure; additionally, long-term channel inhibition may interfere with physiological mechanical signal transduction, leading to an imbalance in mechanical homeostasis.

Third, the physiological relevance of current disease models is limited. Most *in vitro* studies apply homogeneous mechanical stimuli to monolayer cells, which diverges significantly from the heterogeneous, three-dimensional mechanical microenvironment experienced by NP, AF, and CEP cells within the intact disc. While cell stretching, compression, and tunable-stiffness substrate models have powerfully elucidated Piezo1’s role under defined mechanical parameters, they cannot adequately simulate the complex, low-frequency, high-magnitude cyclic loading spectrum of the human upright spine during activities of daily living. Furthermore, rodent models have inherent biomechanical differences from humans, limiting their ability to replicate chronic, low-grade mechanical overloading resulting from prolonged poor posture or occupational strain. Future research necessitates the development of advanced models that better integrate mechanical parameters (intensity, frequency, static/dynamic ratio), cellular heterogeneity, and temporal progression (chronicity). Such models are crucial for more precisely defining the pathological activation threshold of Piezo1 and for validating the long-term efficacy and safety of Piezo1-targeted therapies.

In summary, the therapeutic potential of Piezo1, a key mechanical sensing target in LDD, has been supported by experimental evidence. However, clinical translation requires further exploration of its underlying mechanisms: by integrating *in vivo* and *in vitro* models to elucidate the dynamic regulatory networks within the pathological mechanical microenvironment and overcoming existing technical limitations, precise intervention strategies can be developed for clinical application.
